# Experimental distinction of Autler-Townes splitting from electromagnetically induced transparency using coupled mechanical oscillators system

**DOI:** 10.1038/srep19040

**Published:** 2016-01-11

**Authors:** Jingliang Liu, Hujiang Yang, Chuan Wang, Kun Xu, Jinghua Xiao

**Affiliations:** 1 School of Science, Beijing University of Posts and Telecommunications, Beijing 100876, China; 2 State Key Laboratory of Information Photonics and Optical Communications, Beijing University of Posts and Telecommunications, Beijing, 100876, China

## Abstract

Here we experimentally demonstrated the electromagnetically induced transparency (EIT) and Autler-Townes splitting (ATS) effects in mechanical coupled pendulums. The analogue of EIT and ATS has been studied in mechanical systems and the intrinsic physics between these two phenomena are also been discussed. Exploiting the Akaike Information Criterion, we discern the ATS effect from EIT effect in our experimental results.

Electromagnetically induced transparency (EIT) refers to the coherent interaction of input light with a three-level atomic system^1^. As the optical response is revealed by the interference between two different excitation pathways of the system, the EIT effect eliminates the absorption of input light which opens a narrow transparency window of the output field. During the past decades, EIT effect provides a variety of applications, such as storage of light, slow light propagation and so on. Meanwhile, there is a similar effect on the transmission spectrum with EIT called Aulter-Townes splitting (ATS) which is the field-induced splitting of the optical response and exhibits no interference effects^2^,^3^. This similarity of the spectrum attracts much confusion and discussions on the distinction between EIT and ATS, thus it is becoming an important problem in quantum optics. And recently, various studies have been made in discerning the EIT^4^,^5^,^6^,^7^ effect and ATS^8^,^9^ effect using different systems. Especially, in 2011, Anisimov *et al.* proposed the Akaike Information Criterion (AIC) to discern EIT from ATS in transmission spectrum^10^. Also in the past decades, many studies have been carried out to observe the EIT and ATS effects in different systems. For example in 2002, the classical analogue of EIT effect is observed in mechanical systems^11^. Later, an experimental investigation of the transition between EIT and ATS was carried out by Giner *et al.*^12^, resorting to the method proposed by Anisimov *et al.*^10^. Subsequently, Zhu *et al.* discovered that EIT may occur and a crossover from EIT to ATS is existed in hot molecules^13^, and also in open ladder systems^14^. And Peng *et al.* experimentally studied the analogue of EIT and ATS using coupled whispering gallery mode microtoroid resonators^15^,^16^ which could be further exploited to realize the all-optical devices.

Simple pendulum is a fundamental mechanical system which could be described by simple harmonic oscillation. Based on the research of a simple pendulum, the coupled pendulums are widely studied during the past decades^17^,^18^,^19^ and various experimental phenomena have been found using coupled pendulums. Moreover, the pendulum mechanical system could be used to study complicated problems by multi-pendulums coupling using spring^20^,^21^, a thin horizontal rod^22^, magnets^18^, and so on. And various experimental phenomena could be simulated using these coupled pendulums, such as chaos, synchronization, etc.

The classical analogues of such quantum phenomena also attract much attentions. However, classical analogues of EIT and ATS effects using mechanical systems are not reported by the researchers. Motivated by the all-optical analogues of EIT and ATS using classical coupled systems, we demonstrate the experiment using coupled mechanical systems to discern the ATS effect from EIT effect. Here in this study, the EIT and ATS effects analogues are experimentally observed in mechanically coupled torsion pendulums. And by using the Akaike Information Criterion (AIC), we theoretically discerned ATS effect from EIT effect.

## Results

### Experimental set-up and oscillation spectrums

**Figure 1:**
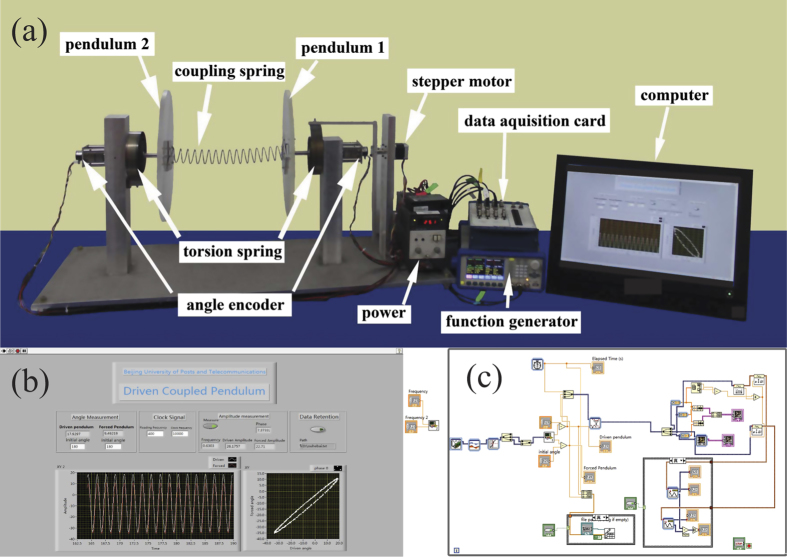
Experimental setup, front panel and block diagram of the program of the system.

Here the experiment setup is shown in Fig. 1. The system consists of two torsion pendulums made of plexiglass with the diameters of 350 mm, the thickness is 10 mm, and the mass is 0.975 kg. As shown in Fig. 1, the two oscillators are coupled by a spring and driven by a stepper motor (42BYG250Bk), here the stepper motor is controlled by the function generator. The data acquisition card (NI, usb6251) is used to collect the oscillation spectrum. The stepper motor is controlled with the function generator through a motor driver (SH2034D). A torsion spring connects the stepper motor to the pendulum 1. In our experiment, the driving frequency is chosen between 0.10–1.20 Hz, and each of the pendulums connect with an angle encoder coaxially. The angle encoder is an 10 bit absolute rotary encoder, and its resolution is 1024 plus per round. We can get the swing angle and the amplitude from the output signal of angle encoder by data acquisition card and VI program. The front panel and block diagram of the VI program are shown also in Fig. 1.

**Figure 2:**
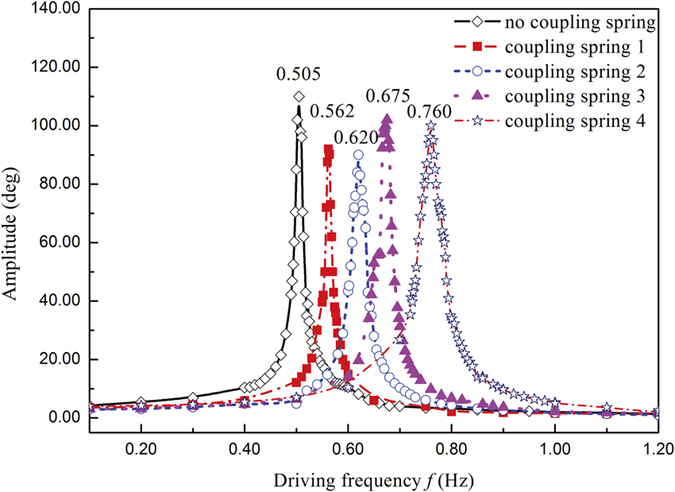
AFC exprimental curves versus the detunings at different freqnencies of the pendulum 1 with fixed pendulum 2. The torsional constant of coupling spring 1–4 are 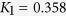 N·m/rad, 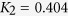 N·m/rad, 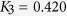 N·m/rad, 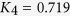 N·m/rad.

The amplitude-frequency characteristics curves (AFC curves) of the pendulum 1 are measured as shown in Fig. 2 when the pendulum 2 is fixed. By cutting off the connection between pendulum 1 and torsion spring, our apparatus can work as a simple driven torsion pendulum. Here we set the driving frequency of 0.10 Hz, and when the pendulum 1 goes into a steady state, we record its amplitude by the VI program. Then by increasing the driving frequency until 1.20 Hz, the steady amplitudes of the pendulum 1 are recorded, and we can get the no coupling spring line. Then, reconnecting the pendulum 1 to torsion spring and fixed the pendulum 2 at its equilibrium position, we can measure the AFC curves with deferent torsion spring, as shown in Fig. 2. By increasing the coupling strength of the coupling spring, we find the increment of the resonance frequency of the pendulum.

### Amplitude-Frequency characteristics curves of coupled pendulums

**Figure 3:**
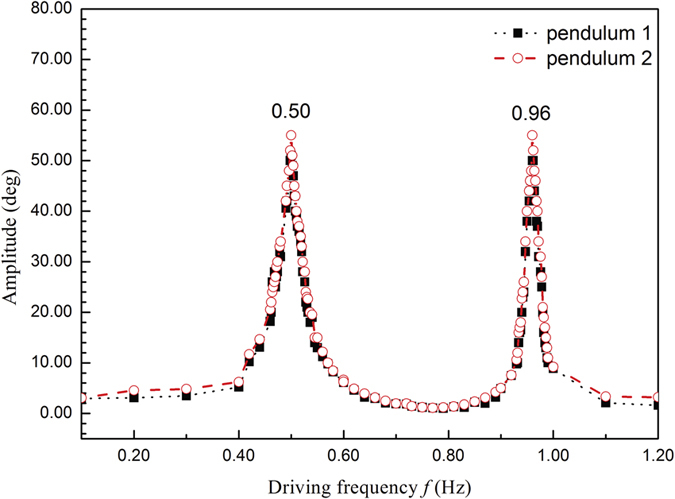
AFC exprimental Curves of the coupled pendulums with coupling spring 4.

To study the coupled system further, we measured the AFC curves of coupled pendulums with the largest coupling strength of spring 4. As shown in Fig. 3, black square dot line refers to pendulum 1, and open circle dash line refers to pendulum 2. There are two resonance peaks 0.50 Hz and 0.96 Hz, and the near zero amplitude point between two resonance peaks is 0.76 Hz (the amplitude is about 1.2°).

**Figure 4:**
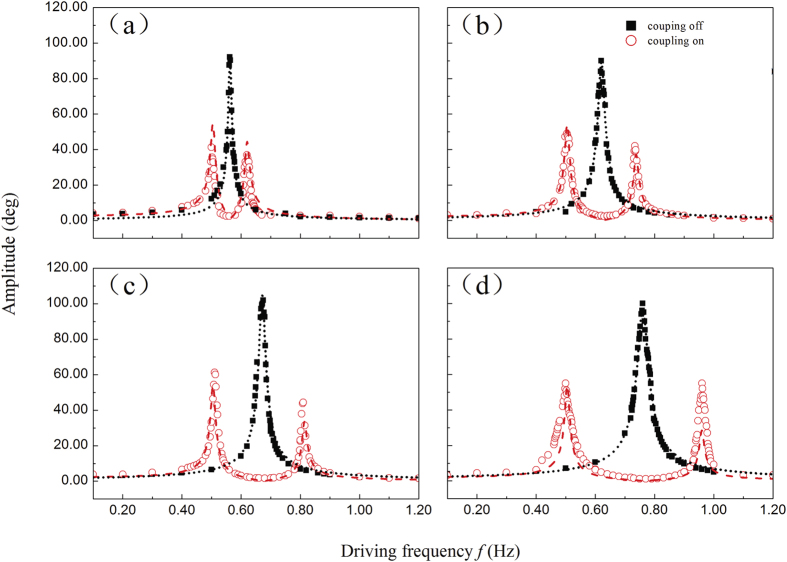
The AFC exprimental and fit curves of the coupled pendulums under different coupling strength. The red dash lines refer to the fit line to Eq. (16), and the black dot line refer to the fit to the amplitude of Eq.(18). (**a**) coupling spring 1, (**b**) coupling spring 2, (**c**) coupling spring 3, (**d**) coupling spring 4.

To study the effect of different coupling strength, we use different coupling springs (spring 1–4 in Fig. 2) to change the coupling strength. And the experiment results are shown in Fig. 4. There is a mode-splitting effect of the absorption spectrum if we turn on the coupling. According to the increment of the coupling strength, the absorption band becomes larger. Meanwhile, the mode splitting effect will be disappeared if we turn off the coupling between the two pendulums. Here the pendulum 1 and 2 are connected with coupling spring, and pendulum 2 is fixed at its equilibrium position. It can be seen obviously that the resonance frequency of the system is increased by coupling spring. And the resonance frequency of a torsion pendulum can be described as


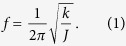


Here, *f*, *k* and *J* are the natural frequency, stiffness and moment of inertia of a torsion pendulum, respectively. In our system, the coupling spring works as an added torsion spring when pendulum 2 is fixed. So the stiffness could be written as 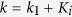, and the moment of inertia is 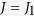.


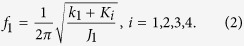


Here, 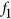, 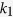 and 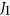 are the natural frequency, stiffness and moment of inertia of pendulum 1, respectively; and 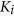 is the stiffness of coupling spring *i*. According to the parameters of pendulum 1, we can calculate its moment of inertia as 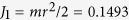 kg·m^2^. And the torsional constant of torsion spring 1 and coupling spring 1–4 could be solved as 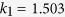 N·m/rad, 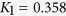 N·m/rad, 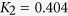 N·m/rad, 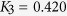 N·m/rad, 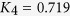 N·m/rad.

### The distinction of ATS from EIT phenomenon using AIC method and analogues with the three-level atomic system

Here we numerically simulated the results of Eq. (16) in the Method part for different coupling strength, and the results are shown in Fig. 4. As shown in the figure, the black dotted line represents the AFC curve of Eq. (18) and the red dash line represents the AFC curve of Eq. (16). We find the EIT and ATS exhibits similar lineshapes in the oscillation spectrum. As shown in our experiment, under the driving of the pump field, the quasi-atomic system shows the EIT effect for the absorption spectrum. It is obvious that such mechanical analogue of EIT phenomenon could be explained as a three-level atomic system: here the three energy levels of the Λ-type atom could be described as the ground state, the excited state and a metastable state. The excitation from the ground state to the excited state and the metastable state to the excited state could be described as the mechanical oscillation of the two oscillators, respectively. Each mechanical system works as the excitation pathways of a three-level atomic system, and the coupling of the two pendulums provides the interaction of the two fields. Due to the destructive interference, the absorption spectral line-shape exhibits a single ‘peak’ at zero frequency detuning. The ATS effect can also exhibit in this system as it attributes to the strong coupling between the two system and splitting the degenerated eigenmodes into two modes.

**Figure 5:**
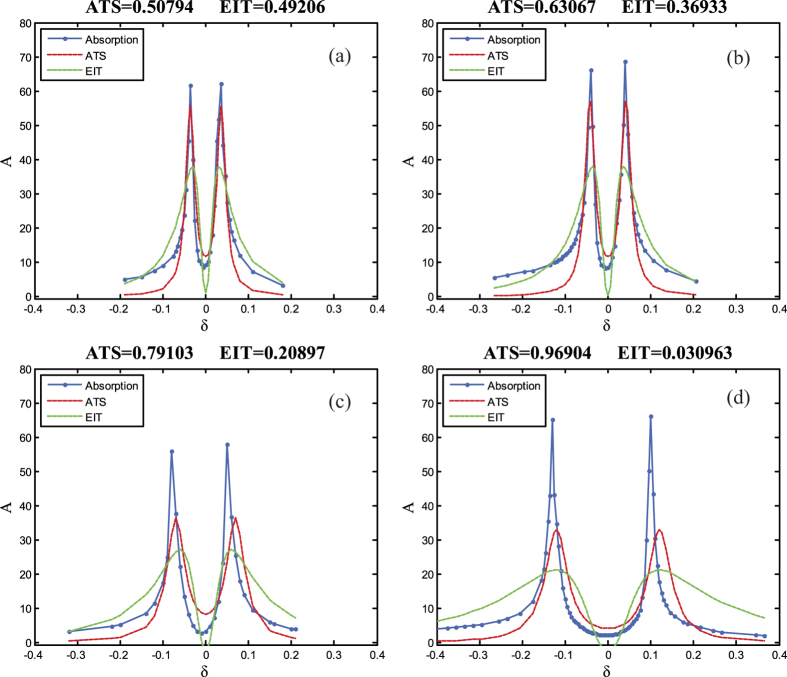
Compare of the simulation and experimental AFC curves of the coupled pendulums under different coupling strength. The blue lines are the experimental results of the amplitude-frequency absorption. The green line and red line are the fitting lines using EIT model and ATS model. And the fitting lines are calculated based on the relative coupling strength as 0.0126 (Fig. 2(a)), 0.0143(Fig. 2(b)), 0.0255(Fig. 2(c)) and 0.0319(Fig. 2(d)), respectively.

As both EIT and ATS effects exhibit similar transparency window in the transmission spectrum, discerning whether a transmission spectrum is the signature of EIT or ATS is an important issue in quantum optics. Here in the experiment, the amplitude-frequency characteristics (AFC) of the coupled pendulums is measured under different coupling strength, and the AFC curves are shown as the blue lines in Fig. 5. To describe the absorption properties of the system, we draw the fitting lines of the experimental results here using EIT and ATS models, respectively. Here we find both EIT and ATS effect in the coupled system. According to refs 10,12, we can get the absorption profiles equations of EIT and ATS as,


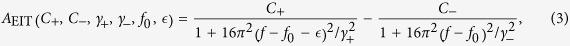



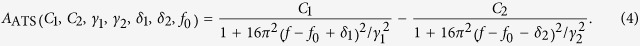


Here, 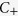, 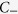, 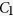, and 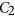 are the amplitudes of the Lorentzian curves; 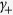, 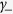, 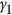, and 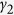 are their respective linewidths; 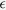, 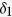, and 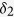 are the shifts from natural frequency.

**Figure 6:**
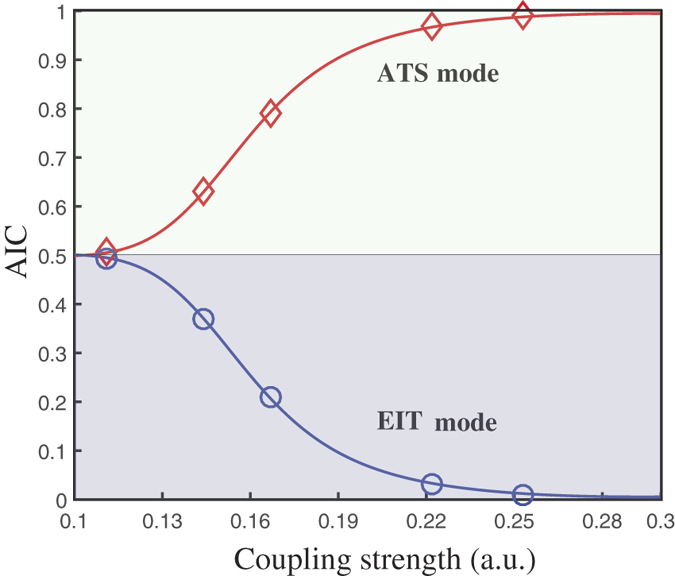
Akaike weights as a function of coupling strength for ATS model(red line) and for EIT model(blue line). Here the two lines are numerically simulated and the points are the experimental data. The relative coupling strength are the numerically solved parameters during the fitting process.

Exploiting the Akaike’s information criterion^10^ to fit our data, we could quantitatively discerning the EIT and ATS models. AIC criterion quantifies the information loss when model 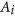 with 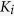 fitting parameters 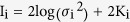 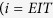 or *ATS*). Here 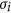 satisfies the relation 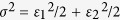, and we use 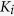 to denote the number of unknown parameters. 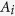 is measured by the relative weights 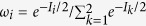, as presented in Fig. 6. Figure 6 describes the system in the ATS domain as the AIC value of the ATS model is larger than the EIT model. By decreasing the coupling strength between the two mechanical oscillators, we found the difference between the AIC values of the ATS model and the EIT model is becoming smaller. And the system could approach the transition point from the ATS domain to the EIT domain. However in our classical system, the coupled oscillation in the undriven system under the condition of weak coupling will be annihilated in the lower frequency domain due to the damping effect. Thus, the transparency window will disappear by continuously decreasing the coupling between the two oscillators, and the EIT domain could hardly be observed.

## Discussion

In conclusion, we have experimentally studied the analogue of EIT effect and ATS effect in coupled mechanical systems and provided a classical analogue of EIT and ATS in this work. We use AIC to test the transmission spectrum of the mechanical system and discerning the ATS effect from EIT effect. By changing the coupling strength, the system could approach the transition point from the ATS domain to the EIT domain, but hardly into the EIT domain. Furthermore, we theoretically analyzed the intrinsic physics of such results in the mechanical system. In addition, our work can be extended to study the parity-time-symmetric system^23^,^24^ by inducing the gain of mechanical oscillation, and the gain assisted nonlinear mechanical effects.

## Method

Here we introduce the theoretical mode of two mechanical oscillators as a three-level atomic system to describe the EIT and ATS effects. The coupled mechanical system could be described using the equations as


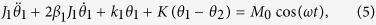



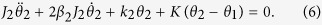


Here 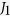 and 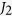 are the momentum of inertia of pendulum 1 and pendulum 2, respectively. 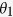 and 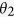 are the swing angle of pendulum 1 and 2; 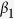 and 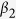 are the friction constant; *K* is the the stiffness of the coupling spring; 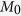 and 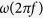 are the amplitude and frequency of the driving force moment, respectively. Then we consider the case 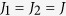, 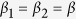, 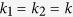, and set the relations 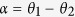, 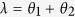, we can get


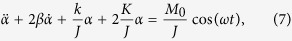



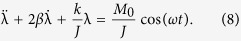


To further simplify the equations, we assume that the frequency 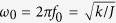, and the parameters are chosen as 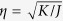, 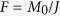, then the equations of the system could be described as


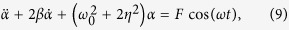



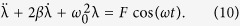


By substituting the steady-state solution to 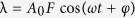 into Eq. (10), we can solve the amplitude and the phase of the mechanical oscillator as


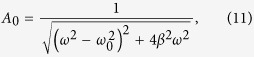



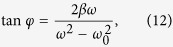


Similarly, by substituting the steady-state solution to 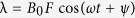 into Eq. (9), the amplitude and phase could be solved as


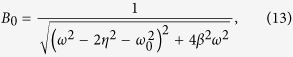



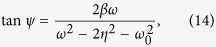


Then, the displacement of the two oscillators could be written as the superposition of the two solutions as


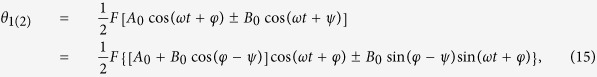


And the amplitudes of the two oscillators 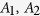 could be expressed as


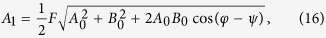



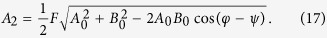


For one oscillator case in this coupled system, we set the pendulum 2 is fixed as 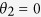, Eq. (5) could be solved as


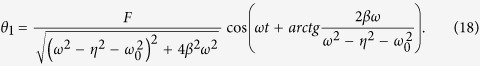


## Additional Information

**How to cite this article**: Liu, J. *et al.* Experimental distinction of Autler-Townes splitting from electromagnetically induced transparency using coupled mechanical oscillators system. *Sci. Rep.* **6**, 19040; doi: 10.1038/srep19040 (2016).
